# Qixian Tongluo Formula promotes the directional differentiation of neural stem cells into neurons via fibrinogen-mediated BMPRI/ID3 signal axis after ischemic stroke

**DOI:** 10.1186/s13023-025-04080-w

**Published:** 2025-10-30

**Authors:** Shengqiang Zhou, Bo Li, Guo Mao, Wen Zeng, Yanjun Chen, Jia Huang, Lingjuan Tan, Dahua Wu, Fang Liu

**Affiliations:** 1 National TCM Master Liu Zuyi Inheritance Studio, Hunan Provincial Hospital of Integrated Traditional Chinese and Western Medicine (The Affiliated Hospital of Hunan Academy of Traditional Chinese Medicine), No. 58, Lushan Road, Yuelu District, Changsha City, Hunan Province 410006 People’s Republic of China; 2 Department of Pediatrics, The First Hospital of Hunan University of Chinese Medicine, Hunan Province, 410007 People’s Republic of China; 3 Key Project Office, Hunan Provincial Hospital of Integrated Traditional Chinese and Western Medicine (The Affiliated Hospital of Hunan Academy of Traditional Chinese Medicine), Hunan Province, 410006 People’s Republic of China; 4 Graduate School, Hunan University of Chinese Medicine, Hunan Province, 410208 People’s Republic of China; 5 Department of Neurology, Hunan Provincial Hospital of Integrated Traditional Chinese and Western Medicine (The Affiliated Hospital of Hunan Academy of Traditional Chinese Medicine), Hunan Province, 410006 People’s Republic of China

**Keywords:** Qixian Tongluo Formula, Neural stem cells, Ischemic stroke, Cell differentiation, Fibrinogen, BMPRI/ID3 signal axis

## Abstract

Promoting nerve regeneration is a crucial approach in treating ischemic stroke, with neural stem cells (NSCs) playing a vital role in this process. Qixian Tongluo Formula (QXTLF) has shown effectiveness in treating ischemic stroke, yet its impact on regulating the directional differentiation of NSCs during ischemic stroke remains unclear. A middle cerebral artery occlusion (MCAO) model was induced in Sprague-Dawley rats, and primary NSCs were isolated from these rats. By gavage QXTLF (7.83 g/kg/d or 31.32 g/kg/d) was administered intragastrically to the MCAO rats. In vitro experiments confirmed the successful isolation of NSCs from rats, capable of differentiating into immature neurons, mature neurons and astrocytes. In vitro, NSCs were exposed to fibrinogen (Fg) and induced by low glucose-oxygen (LGO) conditions to simulate the NSC niche in ischemic stroke. Various assessments were conducted, including neurological score evaluation in MCAO rats, analysis of infarct size using 2,3,5-triphenyltetrazolium chloride (TTC) staining, and examination of the expression of markers like bromodeoxyuridine (BrdU), doublecortin (DCX), microtubule-associated protein 2 (MAP-2), glial fibrillary acidic protein (GFAP), and bone morphogenetic protein type I receptor (BMPRI)/inhibitor of DNA binding 3 (ID3) by immunofluorescence and western blot analysis. The Fg content was quantified by enzyme-linked immunosorbent assay. Results demonstrated that QXTLF reduced cerebral infarction and decreased the neurological score of MCAO rat in a dose-dependent manner. Additionally, QXTLF promoted the directional differentiation of NSCs into neurons within ipsilateral hippocampus following cerebral ischemia. Furthermore, the study revealed that QXTLF facilitated the directional differentiation of LGO-induced NSCs into neurons and implicated Fg in mediating this effect. QXTLF was found to regulate the directional differentiation of LGO-induced NSCs through the Fg-mediated BMPRI/ID3 signal axis, highlighting its role in promoting the differentiation of NSCs into neurons post-ischemic stroke.

## Introduction

Stroke is a leading cause of long-term disability and mortality worldwide, ranking among the most prevalent cerebrovascular diseases [1]. It is generally classified into ischemic and hemorrhagic types, with ischemic stroke accounting for approximately 80% of all cases [2]. Ischemic stroke often leads to significant neurological impairments, including motor, cognitive, and sensory deficits. A pivotal pathological mechanism in ischemic stroke is ischemia/reperfusion (I/R) injury, which drives progressive brain damage through a self-perpetuating cascade of oxidative stress, neuroinflammation, and neuronal apoptosis, thus disrupting neurological recovery [3, 4]. Neurogenesis plays a crucial role in the brain’s self-repair process following injury, with evidence suggesting its potential to mitigate and even reverse neurological damage [5]. Neural stem cells (NSCs) are central to this regenerative process [6]. After ischemic injury, NSCs migrate to the infarcted and peri-infarct regions, where they differentiate into functional neurons, contributing to brain repair [7, 8]. Notably, NSC transplantation has emerged as a promising therapeutic strategy for ischemic stroke [9]. However, the exact mechanisms still remain unclear. A deeper understanding of NSCs differentiation during neural regeneration is essential for identifying novel therapeutic targets.

Fibrinogen (Fg), a crucial protein in fibrin gel formation, plays a significant role in innate immunity, wound healing and inflammatory responses [10]. Under normal physiological conditions, the blood-brain barrier (BBB) effectively excludes Fg from the central nervous system (CNS). However, BBB integrity is compromised during cerebral ischemia, leading to Fg extravasation into the brain parenchyma, where it exacerbates neuroinflammation and neuronal injury [11–14]. For instance, elevated fibrinogen levels in aging mice induced blood-brain barrier dysfunction by downregulating mitochondrial-related proteins and disrupting endothelial cell integrity [15]. Furthermore, Fg impaired neonatal cerebellar development by inhibiting Sonic Hedgehog signaling and promoting neuroinflammation after blood-brain barrier disruption [14]. Additionally, Fg promotes differentiation of neural stem/precursor cells into astrocytes via bone morphogenic protein (BMP) receptor signaling, thus potentially suppressing neuronal differentiation [16]. However, the precise role of Fg in ischemic stroke remains unclear. Recent studies highlight the involvement of bone morphogenetic protein type I receptor (BMPRI)/inhibitor of DNA binding 3 (ID3) axis in cell proliferation and differentiation across different diseases. For instance, bone morphogenetic protein 4 (BMP4) enhanced the proliferation of human Sertoli cells by activating Smad1/5 signaling pathway and the ID2/3 pathway in the context of azoospermia [17]. Furthermore, ID2/3 plays a crucial role in modulating neurogenesis and the differentiation of NSCs [18]. Nevertheless, the specific role of BMPRI/ID3 axis in NSCs remains underexplored.

Qixian Tongluo Formula (QXTLF) is a traditional Chinese herbal medicine developed by Professor Liu Zuyi, a renowned master of traditional Chinese medicine (TCM), for treating cerebral infarction and its sequelae. The formula is composed of medicinal plants, including astragalus root, epimedium, salvia, polygonum multiflorum, wolfberry, kudzu root, leech and hawthorn [19]. The principles of TCM that underpin QXTLF, such as promoting blood circulation and replenishing Qi, originated in northern China thousands of years ago. Professor Liu developed this formula by integrating these ancient principles with modern clinical insights. Over time, it has been refined for modern clinical use, especially for treating cerebral infarction syndrome. Our previous research showed that QXTLF improved the clinical outcomes in patients with cerebral infarction [20]. Another study revealed that QXTLF enhanced the regeneration of endogenous neural stem cells in rats with cerebral ischemia, particularly during the recovery phase [21]. However, its regulatory mechanisms in ischemic stroke have not yet been fully addressed. We hypothesized that QXTLF may mitigate the extent of ischemic stroke by modulating the Fg-mediated BMPRI/ID3 signaling axis.

Based on our previous studies, the low glucose-oxygen (LGO)-induced neuronal injury model can simulate the low perfusion microenvironment of neurons surrounding cerebral infarction in ischemic stroke [22]. Therefore, this study utilized both the MCAO animal model and the LGO-induced cell model to explore the mechanism by which QXTLF regulated the directional differentiation of NSCs to improve ischemic stroke. Our findings may provide promising targets for ischemic stroke treatment.

## Materials and methods

### Animals and QXTLF

Male Sprague-Dawley (SD) rats (weighing 300 ± 20 g) were purchased from SJA Laboratory Animal Company (Hunan, China). The rats were housed in a light-controlled room on a 12 h-light/12 h-dark cycle at 25℃ with free access to water and food. All experimental procedures were approved by the Experimental Animal Ethics Committee of Hunan Academy of Chinese Medicine (NO. 2022-0083) and were conducted in accordance with institutional guidelines.

QXTLF is a traditional Chinese herbal medicine composed of the following ingredients: astragalus (30 g), xianling spleen (15 g), salvia miltiorrhiza (30 g), pueraria lobata (30 g), lycium barbarum (30 g), processed reynoutria multiflora (30 g), leech (9 g) and hawthorn (15 g). The chemical composition of QXTLF was analyzed using ultra-performance liquid chromatography coupled with quadrupole time-of-flight mass spectrometry (UPLC-Q-TOF/MS).

### Study design

SD rats were randomly allocated into four groups: sham, middle cerebral artery occlusion (MCAO), MCAO + 7.83 g/kg/d QXTLF (MCAO + QXTLF-L), and MCAO + 31.32 g/kg/d QXTLF (MCAO + QXTLF-H). MCAO rats were administered QXTLF via gavage at different doses, while the sham group underwent the same procedure without MCAO. QXTLF was administered daily for 21 days. Additionally, the rats were injected intraperitoneally with 5-bromodeoxyuridine (BrdU) at a dosage of 50 mg/kg/d once a day from 5 d after MCAO procedure, with the BrdU injection continued for 4 days.

### MCAO rat model

Focal cerebral ischemia was induced using the MCAO model as previously described [23]. Briefly, SD rats were anesthetized with 0.5-3% isoflurane and fixed on a heating table. The internal carotid artery (ICA), right external carotid artery (ECA), and right common carotid artery (CCA) were exposed and isolated. A filament was inserted into the ICA via the ECA to occlude blood flow, and the distal CCA was ligated. The successful establishment of MCAO model was confirmed using the Zea-Longa scoring criteria [24].

### Neurological function evaluation

The neurological deficits were evaluated using the modified neurological severity score (mNSS) [25]. The mNSS encompasses evaluations of motor function (6 points), sensory function (2 points), balance test function (6 points), and reflexes/abnormal activity (4 points). The total score ranges from 0 (normal) to 18 (maximal deficit), with higher scores indicating greater impairment.

*2*,*3*,*5-triphenyltetrazolium chloride (TTC) staining*.

TTC staining, based on the activity of succinate dehydrogenase, is a widely used histochemical technique to assess cerebral ischemic injury [26, 27]. This technique generates a red formazan product through enzyme-catalyzed reactions in the mitochondria of living cells, while ischemic areas appear pale due to the lack of enzyme activity, creating a clear contrast. After euthanasia, rat brains were swiftly extracted and stored at -70℃. The brains were then sliced into 2-mm-thick coronal slices, incubated in 2% TTC at 37℃ for 30 min, and fixed in 4% paraformaldehyde for 12 h. Infarction and hemisphere areas of the sections were analyzed using the program ImageJ (1.8.0, National Institute of Health, USA), and cerebral infarction size was calculated and quantified.

### Cell isolation and culture

NSCs were isolated from the hippocampus of 14-day-old pregnant SD rats [28]. Bilateral hippocampal tissues were dissected, digested in 0.125% trypsin solution for 20 min at 37℃. Cell suspensions were seeded into 6-well plates (1 × 10^5^ cells/mL) and cultured in serum-free Dulbecco’s Modified Eagle Medium/Nutrient Mixture F-12 (DMEM/F12) supplemented with 10% fetal bovine serum (FBS), 20 ng/mL recombinant rat basic fibroblast growth factor, 20 ng/mL recombinant rat epidermal growth factor, 2% B27, 100 U/mL penicillin, and 100 mg/mL streptomycin (Gibco, USA). Neurospheres were passaged every 7–10 days, and NSCs from the third passage were used for subsequent experiments.

### Low glucose-oxygen (LGO) model and treatment

The LGO model was established as previously described [22]. NSCs were cultured in low glucose medium (5 mmol/L glucose) under 2% O_2_ conditions at 37℃ for 48 h in a hypoxic chamber. Control cells were maintained under normoxic conditions. LGO-induced NSCs were treated with 1 mg/mL or 2.5 mg/mL Fg and either serum containing QXTLF at 10% concentration (QXTLF-LS) or 20% concentration (QXTLF-HS) for 3 days. Low concentration of 1 mg/mL Fg simulated the environment during cerebral infarction, while the higher concentration of 2.5 mg/mL Fg simulated the deposition of Fg.

### Immunofluorescence

The expression levels of BrdU, doublecortin (DCX), microtubule-associated protein 2 (MAP-2), glial fibrillary acidic protein (GFAP) and Nestin in NSCs were analyzed using an immunofluorescence assay. NSCs were fixed in 4% paraformaldehyde, permeabilized with 0.3% Triton X-100, and blocked with 10% bovine serum albumin (BSA). Primary antibodies against BrdU (ab152095, 1/100, Abcam, USA), DCX (ab207175, 1/250, Abcam), MAP-2 (ab183830, 1/500, Abcam), GFAP (ab7260, 1/5000, Abcam), and Nestin (ab92391, 1/250, Abcam) were applied overnight, followed by Goat Anti-Rabbit IgG H&L (Alexa Fluor^®^ 594) secondary antibody (ab150080, 1/200, Abcam) and counterstained with 4’,6-diamidino-2-phenylindole (DAPI). Images were acquired using a confocal microscope.

### Quantitative real time PCR (qRT-PCR)

Total RNA was extracted from neural spheres using Trizol reagent (Tiangen Biotech, China), reverse-transcribed into cDNA using the PrimeScript™ RT Reagent Kit (TaKaRa, Dalian, China), and amplified using SYBR Green Master Mix kit (Solarbio Science & Technology Co., Ltd., China) on an Exicycler™ 96 system (Bioneer Corporation, Korea). The primer sequences were designed as follows: Sox2 (F) 5’-CACAACTCGGAGATCAGCAA-3’, (R) 5’-CGGGGCCGGTATTTATAATC-3’; Nestin (F) 5’-GATCTAAACAGGAAGGAAATCCAGG-3’, (R) 5’-TCTAGTGTCTCATGGCTCTGGTTTT-3’; GAPDH (F) 5’-TATGATGATATCAAGAGGGTAGT-3’, (R) 5’-TGTATCCAAACTCATTGTCATAC-3’. Data analysis was performed using the 2^–ΔΔCT^ methods [ΔCT = CT(Target gene)-CT(GAPDH), ΔΔCT = ΔCT(Day 7)-ΔCT(Day 3)], with GAPDH serving as the internal control.

### Western blotting

Proteins were extracted from NSCs, and the concentrations of GFAP, DCX, MAP-2, BMPRI, Smad1/5/8 and ID3 were quantified using the Bicinchoninic Acid (BCA) kit (Sigma Aldrich). Equal volumes of protein were loaded onto sodium dodecyl sulfate polyacrylamide gel electrophoresis (SDS-PAGE) and transferred onto polyvinylidene fluoride (PVDF) membranes. After blocking with 5% non-fat dry milk, the membranes were probed with primary antibodies against GFAP (ab7260, 1/10000, Abcam), DCX (ab18723, 1/1000, Abcam), MAP-2 (ab32454, 1/1000, Abcam), BMPRI (ab130206, 1/500, Abcam), Smad1/5/8 (MBS619767, 1/2000, MybioSource) and ID3 (ab86017, 1/1000, Abcam) at 4℃ overnight. The membranes were then incubated with secondary antibodies, including HRP anti-Rabbit IgG antibody (ab288151, 1/2000, Abcam) or Goat Anti-Mouse IgG H&L preadsorbed (ab7063, 1/2000, Abcam) for 1 h at room temperature. Signals were detected using the Super Signal West Pico Chemiluminescent Substrate kit (Thermo Fisher Scientific, Inc., USA) and protein bands were quantified using Image-Pro Plus software (Media Cybernetics, Inc., USA).

### Enzyme linked immunosorbent assay (ELISA)

Fg levels in LGO-induced NSCs were detected using ELISA method. A 10% cell homogenate was prepared using radio immunoprecipitation assay (RIPA) buffer, followed by centrifugation at 4℃ for 15 min. The Fg concentration in the supernatant was quantified using a commercial ELISA kit (MBS2515916, detection range 62.5–4000 ng/mL, MybioSource).

### Statistical analysis

All data were presented as mean ± standard deviation (SD) and analyzed using GraphPad Prism 7.0. Comparisons among three or more groups were performed using one-way analysis of variance (ANOVA) followed by Tukey’s post hoc test. Comparisons between two groups were made using Student’s *t*-test. *P* < 0.05 was considered to be statistically significant. All experiments were conducted at least three times.

## Results

### Chemical composition of QXTLF

**Fig. 1:**
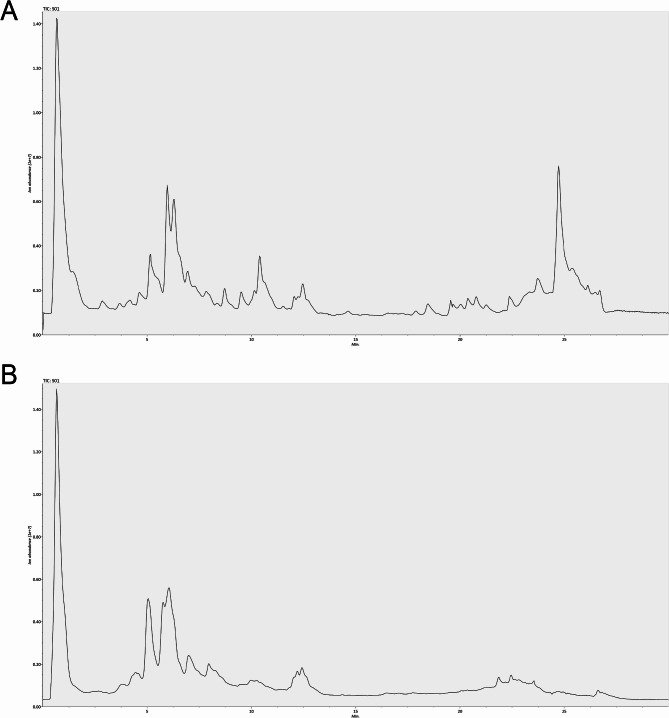
UPLC-Q-TOF/MS total ion chromatogram of QXTLF. (**A**) Positive ion mode. (**B**) Negative ion mode

The retention times and mass spectrometry data of QXTLF compounds were analyzed using UPLC-Q-TOF/MS. Total ion chromatograms of QXTLF (Fig. 1) identified 15 compositions, including ononin, formononetin, and puerarin. A detailed list of these compounds is shown in Table 1. These findings highlight that flavonoids, including ononin, formononetine, puerarin, icariin, glycitin, kaempferol-3-O-glucoside, quercetin, genistein, daidzin, caffeic acid and catechin, were identified as the predominant chemical constituents of QXTLF.

**Table 1:** Information of main compounds of QXTLF

No	Positive ion mode			Negative ion mode			Molecular weight	Molecular formula	Compound name	Attribution
	RT (min)	adduct	m/z	RT (min)	adduct	m/z				
1	12.222	/	431.13541	10.285	[M-H]-	429.12497	430.405	C_22_H_22_O_9_	ononin	*Astragalus mongholicus* Bunge
2	14.596	[M + Na]+	269.08383	14.345	[M-H]-	267.06747	268.264	C_16_H_12_O_4_	Formononetine	*Astragalus mongholicus* Bunge
3	5.96	[M + Na]+	417.12106	5.745	[M-H]-	415.00259	432.381	C_21_H_20_O_9_	puerarin	*Pueraria lobata (Willd.) Ohwi*
4	12.455	[M + Na]+	677.25531	16.007	[M-H]-	675.22974	676.73	C_33_H_40_O_15_	Icariin	*Epimedium brevicornum Maxim*
5	7.811	[M + Na]+	447.13361	5.412	[M-H]-	446.44	480.1575	C_22_H_22_O_10_	Glycitin	*Pueraria lobata (Willd.) Ohwi*
6	8.684	/	471.0954	13.19	[M-H]-	593.13	448.41	C_21_H_20_O_11_	Kaempferol-3-O-glucoside	*Astragalus mongholicus* Bunge
7	8.819	/	303.05237	0.851	[M-H]-	603.08337	302.25	C_15_H_10_O_7_	Quercetin	*Astragalus mongholicus* Bunge
8	8.363	/	271.06137	8.38	[M-H]-	269.04919	270.25	C_15_H_10_O_5_	Genistein	*Salvia miltiorrhiza Bge.*
9	6.944	[M + Na]+	417.12106	6.958	[2 M-H]-	831.2149	416.41	C_21_H_20_O_9_	Daidzin	*Pueraria lobata (Willd.) Ohwi*
10	11.001	/	181.05237	1.293	[M-H]-	179.03543	180.17	C_9_H_8_O_4_	Caffeic acid	*C. pinnatifida Bge.*
11	3.826	/	163.03981	5.336	[M-H]-	359.07724	360.34	C_18_H_16_O_8_	Rosmarinic acid	*Salvia miltiorrhiza Bge.*
12	15.687	/	807.45465	15.647	[M-H]-	843.48175	784.97	C_41_H_68_O_14_	Astragaloside IV	*Astragalus mongholicus* Bunge
13	18.02	/	271.06369	5.344	[M-H]-	269.04456	270.25	C_15_H_10_O_5_	Emodin	*Polygonum multiflorum Thunb*
14	10.381	/	313.06744	6.074	[M-H]-	289.07483	290.29	C_15_H_14_O_6_	catechin	*Lycium barbarum L.*
15	22.115	/	439.36121	21.883	[M-H]-	455.35324	456.78	C_30_H_48_O_3_	Ursolic acid	*Salvia miltiorrhiza Bge.*

### QXTLF attenuates cerebral infarction and promotes the directional differentiation of NSCs into neurons in MCAO rats

**Fig. 2:**
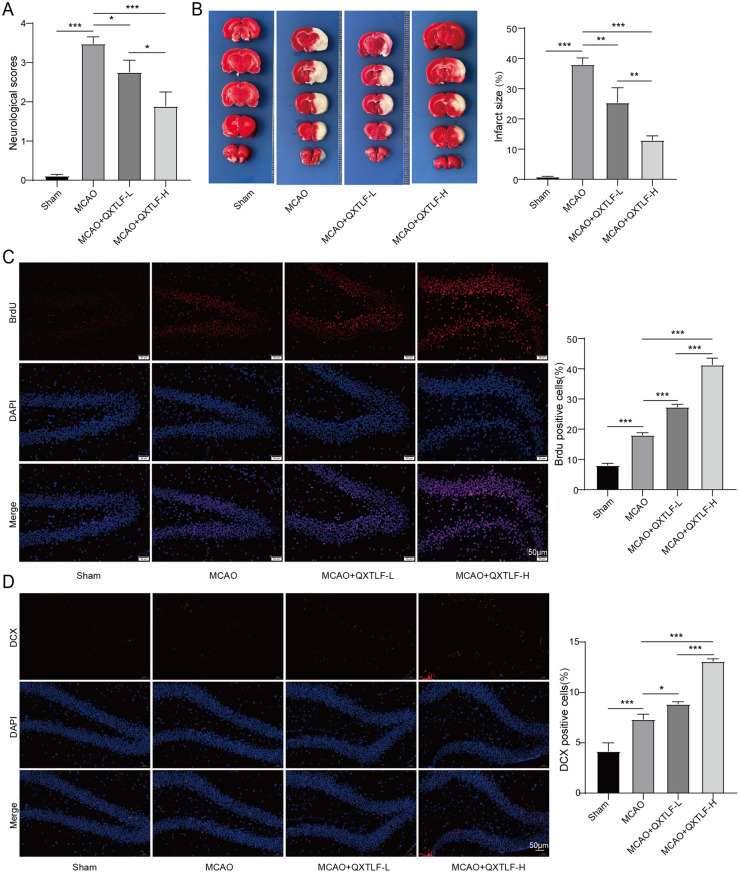

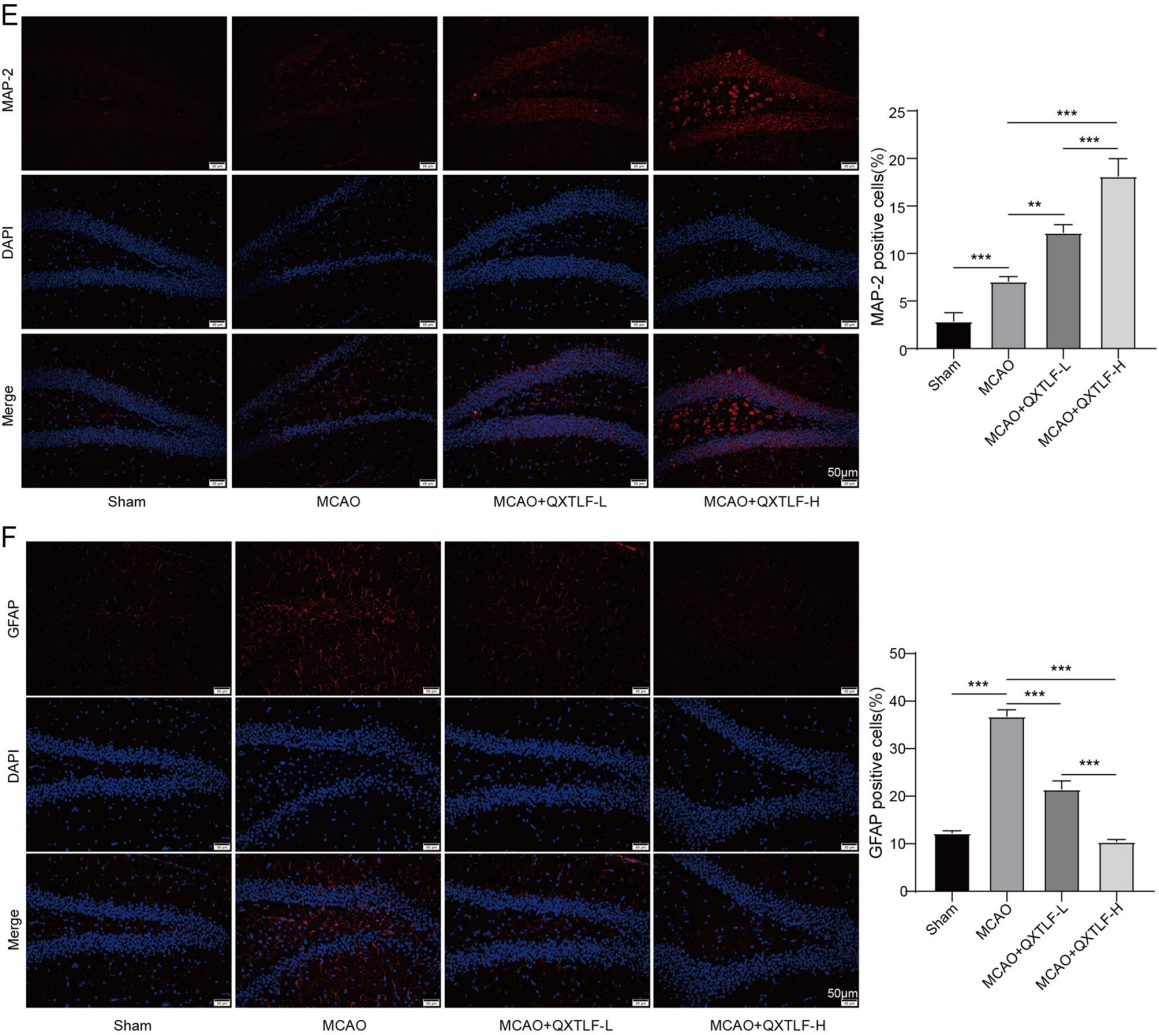
The effect of QXTLF on cerebral infarction and promotion of the directional differentiation of NSCs into neurons in MCAO rats. (**A**) A MCAO rat model was established and MCAO rats were administered QXTLF intragastrically at a dose of 7.83 g/kg/d (QXTLF-L) or 31.32 g/kg/d (QXTLF-H). The neurological score was quantitation on day 21 after MCAO. (**B**) The cerebral infarction was analyzed by TTC staining in rats. (**C**) Representative image and the quantitation of BrdU positive cells in the hippocampi of rats. Scale bar: 50 μm. (**D**) Representative image and the quantitation of DCX positive cells in the subependymal zone of rats. Scale bar: 50 μm. (**E**) Representative image and the quantitation of MAP-2 positive cells in the hippocampi of rats. Scale bar: 50 μm. (**F**) Representative image and the quantitation of GFAP positive cells in the hippocampi of rats. Scale bar: 50 μm. Data are presented as mean ± SD. *P < 0.05, **P < 0.01, ***P < 0.001

Since QXTLF has been reported to improve recovery after cerebral infarction [29], a MCAO rat model was established to investigate its effects. MCAO rats received oral administration of QXTLF at 7.83 g/kg/d or 31.32 g/kg/d. As depicted in Fig. 2A, MCAO rats exhibited significantly higher mNSS scores than sham controls. However, QXTLF treatment reduced neurological deficits in a dose dependent manner in MCAO rats. Additionally, TTC staining demonstrated significantly larger infarct volumes in MCAO group compared with sham controls. Conversely, QXTLF administration significantly attenuated cerebral infarction in MCAO rats (Fig. 2B). Considering the pivotal role of neurogenesis in stroke recovery [30], we hypothesized that QXTLF promotes NSC differentiation into neurons. To verify this, we assessed the proliferation and differentiation of NSCs in the ipsilateral hippocampus of MCAO rats. Quantitative analysis demonstrated that the number of BrdU-positive cells was increased in the MCAO group compared to the sham group. Furthermore, QXTLF treatment enhanced the number of BrdU-positive cells in a dose-dependent manner (Fig. 2C). In addition, the immature neuron marker DCX showed elevated expression in MCAO rats compared to sham group. QXTLF administration enhanced the number of DCX-positive immature neurons in MCAO rats (Fig. 2D) in a dose dependent manner. A similar dose-dependent increase was observed for the mature neuron marker MAP-2 in MCAO rats (Fig. 2E). Immunofluorescence staining also revealed that QXTLF reduced the increased content of GFAP-positive cells in MCAO rats (Fig. 2F). These findings collectively suggest that QXTLF protected against cerebral infarction in MCAO rats but also promoted the directional differentiation of NSCs into neurons. Considering the therapeutic potential of NSC differentiation for ischemic stroke [31], we further explore whether QXTLF could directly modulate NSC differentiation in vitro.

### Identification and differentiation of NSCs

**Fig. 3:**
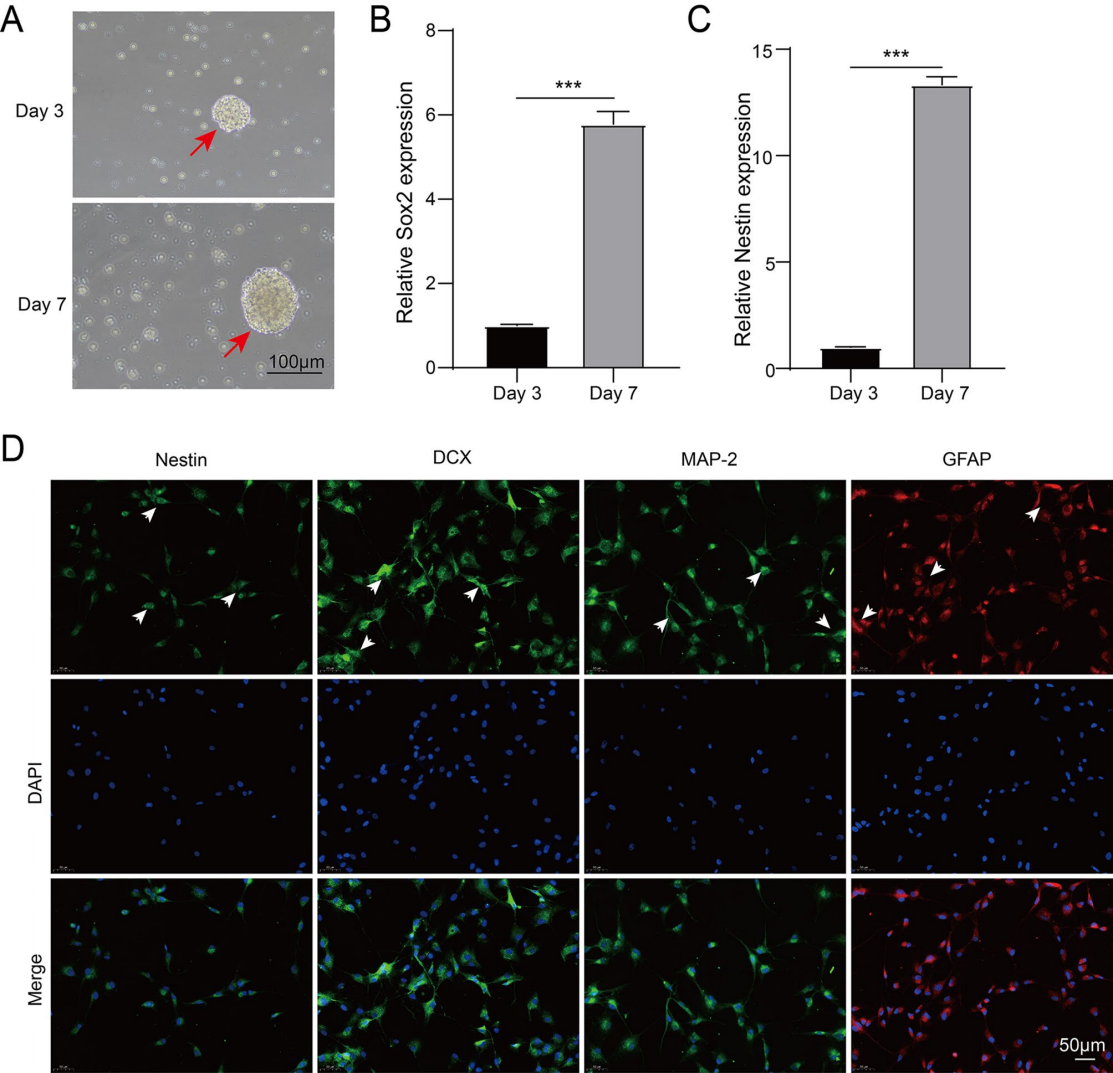
Cell culture, identification and differentiation of NSCs. (**A**) Cell morphology of NSCs on day 3 and day 7 was observed under the microscope. (**B**) The expressions of NSCs marker Sox2 on day 3 and day 7 were determined by qRT-PCR. (**C**) The expressions of NSCs marker Nestin on day 3 and day 7 were determined by qRT-PCR. (**D**) Fluorescence images of Nestin, DCX, MAP-2 and GFAP in NSCs. Data are presented as mean ± SD. ***P < 0.001

NSCs were isolated from the hippocampus of pregnant female rats at day 14. Over time, NSCs gradually proliferated and formed neural spheres of varying diameters. The morphology of NSCs on day 3 and day 7 is shown in Fig. 3A. On day 7, NSCs exhibited significantly higher expression of Sox2 and Nestin compared to day 3 (Fig. 3B-C). Immunofluorescence images confirmed positive expressions of Nestin, DCX, MAP-2 and GFAP in the purified NSC cultures, validating their ability to differentiate into immature neurons, mature neurons and astrocytes (Fig. 3D). These results confirmed the successful isolation of NSCs.

### QXTLF promotes the directional differentiation of LGO induced NSCs into neurons

**Fig. 4:**
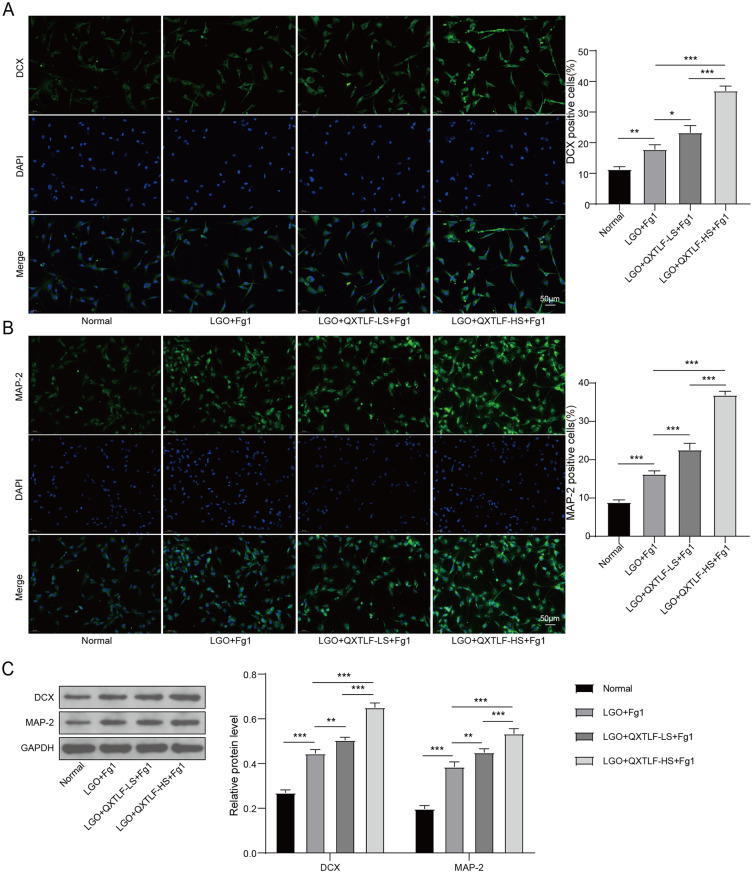

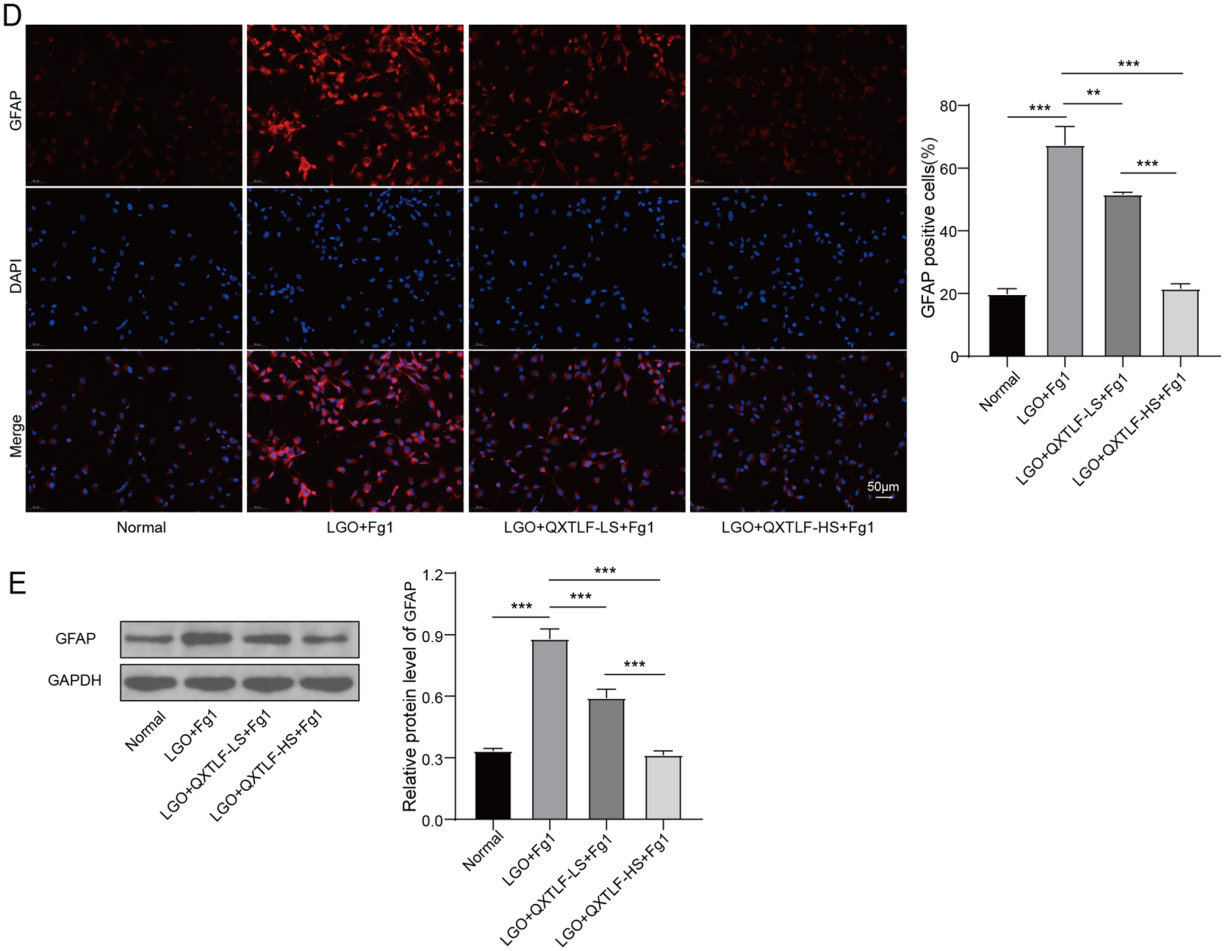
The effect of QXTLF on promoting the directional differentiation of LGO induced NSCs into neurons. (**A**) After exposing to exogenous Fg (1 mg/mL), NSCs were induced by LGO to establish a cell model of injury. After intragastric administration of QXTLF, drug-containing serum of QXTLF was collected from mouse. LGO induced NSCs were treated with drug-containing serum of QXTLF at 10% concentration (QXTLF-LS) or 20% concentration (QXTLF-HS) for 3 d. Fluorescence image and the number of DCX positive cells. Scale bar: 50 μm. (**B**) Fluorescence image and the number of MAP-2 positive cells. Scale bar: 50 μm. (**C**) Protein expressions of DCX and MAP-2 were determined by western blot. (**D**) Fluorescence image and the number of GFAP positive cells. Scale bar: 50 μm. (**E**) Protein expression of GFAP was detected by western blot. Data are presented as mean ± SD. *P < 0.05, **P < 0.01, ***P < 0.001

Based on results obtained from the animal model, we established an in vitro cell culture model to further investigate the effects of QXTLF on the directional differentiation of LGO-treated NSCs into neurons. To simulate in vitro injury, NSCs were exposed to exogenous serum Fg (1 mg/mL) and treated with LGO. After intragastric administration of QXTLF, serum containing QXTLF was collected from the rats for subsequent analysis. LGO-induced NSCs were then treated with the collected serum at two concentrations, 10% (QXTLF-LS) and 20% (QXTLF-HS). LGO exposure increased the populations of DCX-positive cells compared to the normal group. Notably, treatment with serum containing QXTLF led to a dose-dependent enhancement in the number of DCX-positive cells in LGO-treated NSCs, suggesting that QXTLF promoted the directional differentiation of NSCs into immature neurons (Fig. 4A). A similar trend was observed for MAP-2-positive cells, proving that QXTLF accelerated the directed differentiation of NSCs into mature neurons (Fig. 4B). These observations were further supported by western blot analysis of DCX and MAP-2 expression levels (Fig. 4C). Furthermore, the elevated population of GFAP-positive cells was obviously reduced in a dose-dependent manner by serum containing QXTLF (Fig. 4D). Corresponding changes in GFAP protein expression were confirmed by western blot analysis (Fig. 4E). These findings suggested that QXTLF acted at the cellular level to enhance NSC differentiation into neurons under LGO induction, consistent with the results observed in the animal model.

### QXTLF regulates the directional differentiation of LGO induced NSCs through reducing Fg deposition

**Fig. 5:**
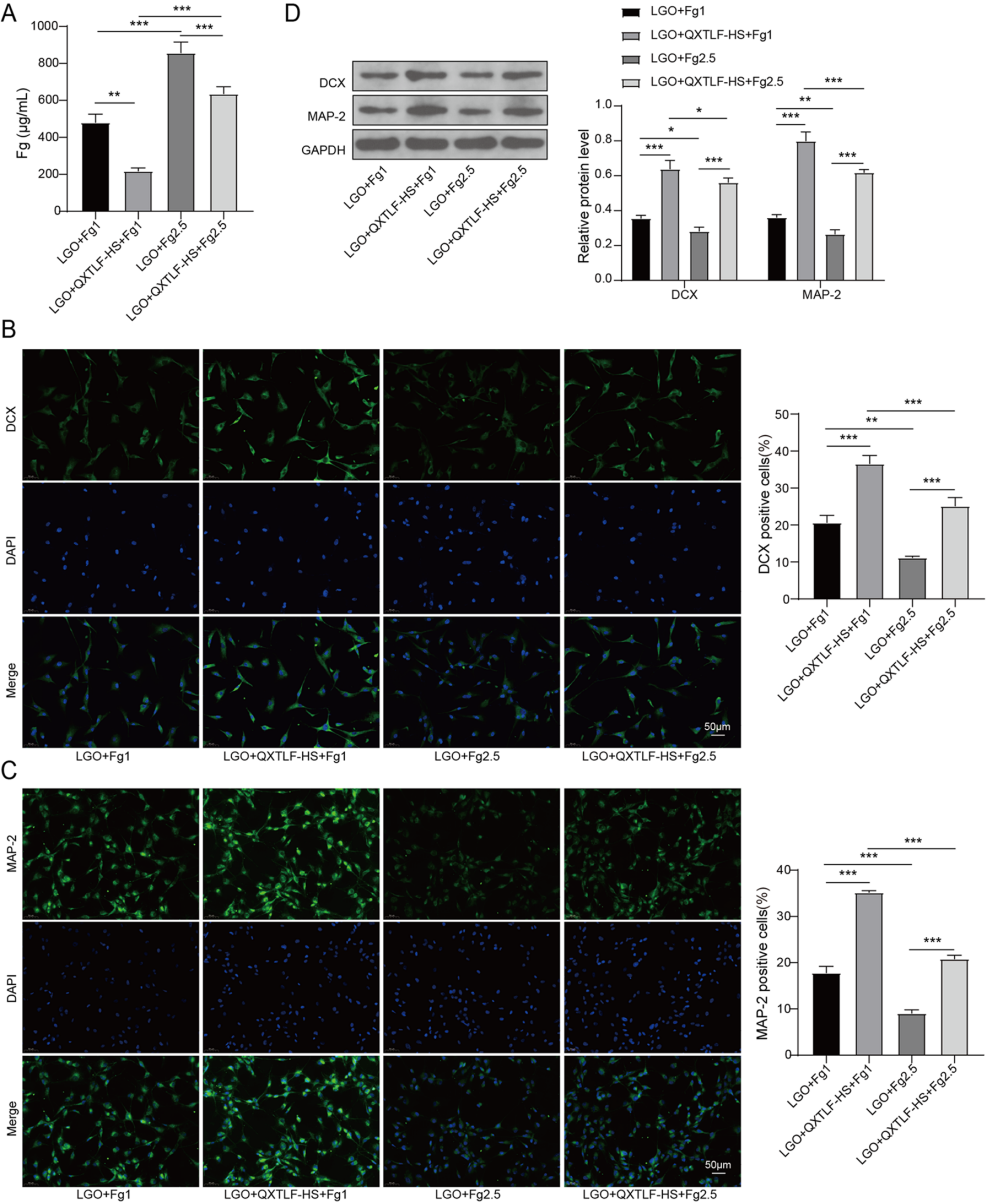

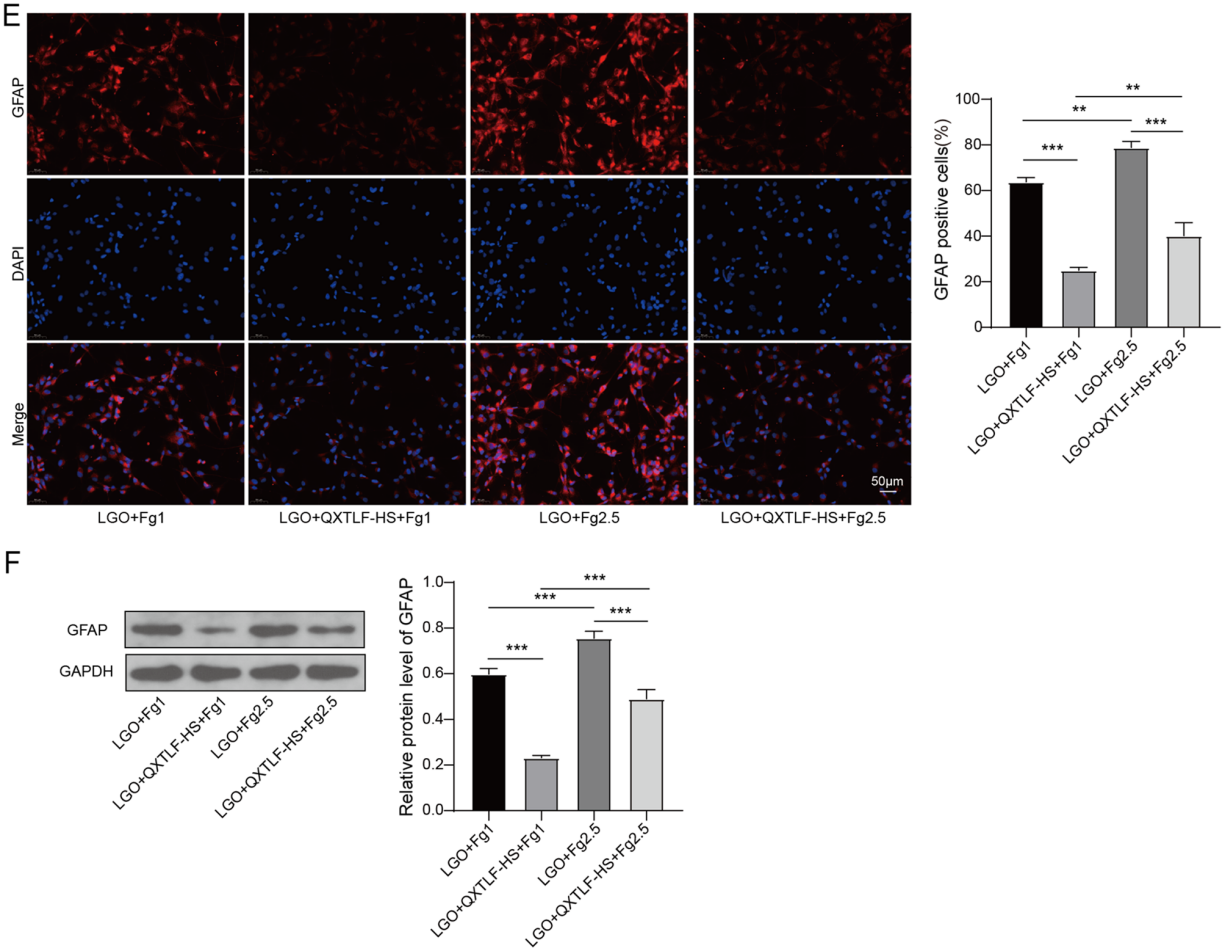
The effect of QXTLF on the regulation of the directional differentiation of LGO induced NSCs through reducing Fg deposition. (**A**) LGO induced NSCs were exposed to exogenous Fg at concentration of 1 mg/mL (Fg1) or 2.5 mg/mL (Fg2.5), respectively. Subsequently, NSCs were treated with drug-containing serum of QXTLF at 20% concentration (QXTLF-HS). Fg content was detected using ELISA. (**B**) Representative image and percentage of DCX positive cells. Scale bar: 50 μm. (**C**) Representative image and percentage of MAP-2 positive cells. Scale bar: 50 μm. (**D**) Protein levels of DCX and MAP-2 were analyzed using western blot. (**E**) Representative image and percentage of GFAP positive cells. Scale bar: 50 μm. (**F**) Protein level of GFAP was determined using western blot. Data are presented as mean ± SD. *P < 0.05, **P < 0.01, ***P < 0.001

Although QXTLF was shown to promote the differentiation of LGO-induced NSCs into neurons, the underlying mechanism remains unclear. To investigate the mechanism by which QXTLF regulates LGO-induced NSCs, we exposed NSCs to exogenous Fg at concentrations of 1 mg/mL (Fg1) or 2.5 mg/mL (Fg2.5) to mimic Fg deposition. High-dose Fg increased the Fg content in LGO-induced NSCs by approximately two-fold compared to low-dose Fg under LGO conditions. Moreover, high-dose Fg reversed the down-regulatory effect of serum containing QXTLF on Fg content in LGO-induced NSCs (Fig. 5A). We then analyzed the impact of Fg on the differentiation ability of LGO-induced NSCs. High-dose Fg significantly reduced the number of DCX-positive cells, counteracting the promoting effect of QXTLF-containing serum on DCX-positive cells (Fig. 5B). High-dose Fg also reduced the content of MAP-2-positive cells in LGO- and QXTLF-treated NSCs (Fig. 5C). Protein expression analysis of DCX and MAP-2 confirmed that serum containing QXTLF activated the differentiation ability of NSCs into neurons, however, this effect was suppressed by high-dose Fg (Fig. 5D). Furthermore, the level of GFAP-positive cells was notably elevated by high-dose Fg treatment compared to NSCs treated with low-dose Fg. Additionally, high-dose Fg reversed the suppressive effect of serum containing QXTLF on GFAP-positive cells (Fig. 5E), and western blot analysis of GFAP supported these findings (Fig. 5F). In summary, QXTLF regulated the directional differentiation of LGO-induced NSCs by reducing Fg deposition.

### QXTLF regulates the directional differentiation of LGO induced NSCs through Fg-mediated BMPRI/ID3 signal axis

**Fig. 6:**
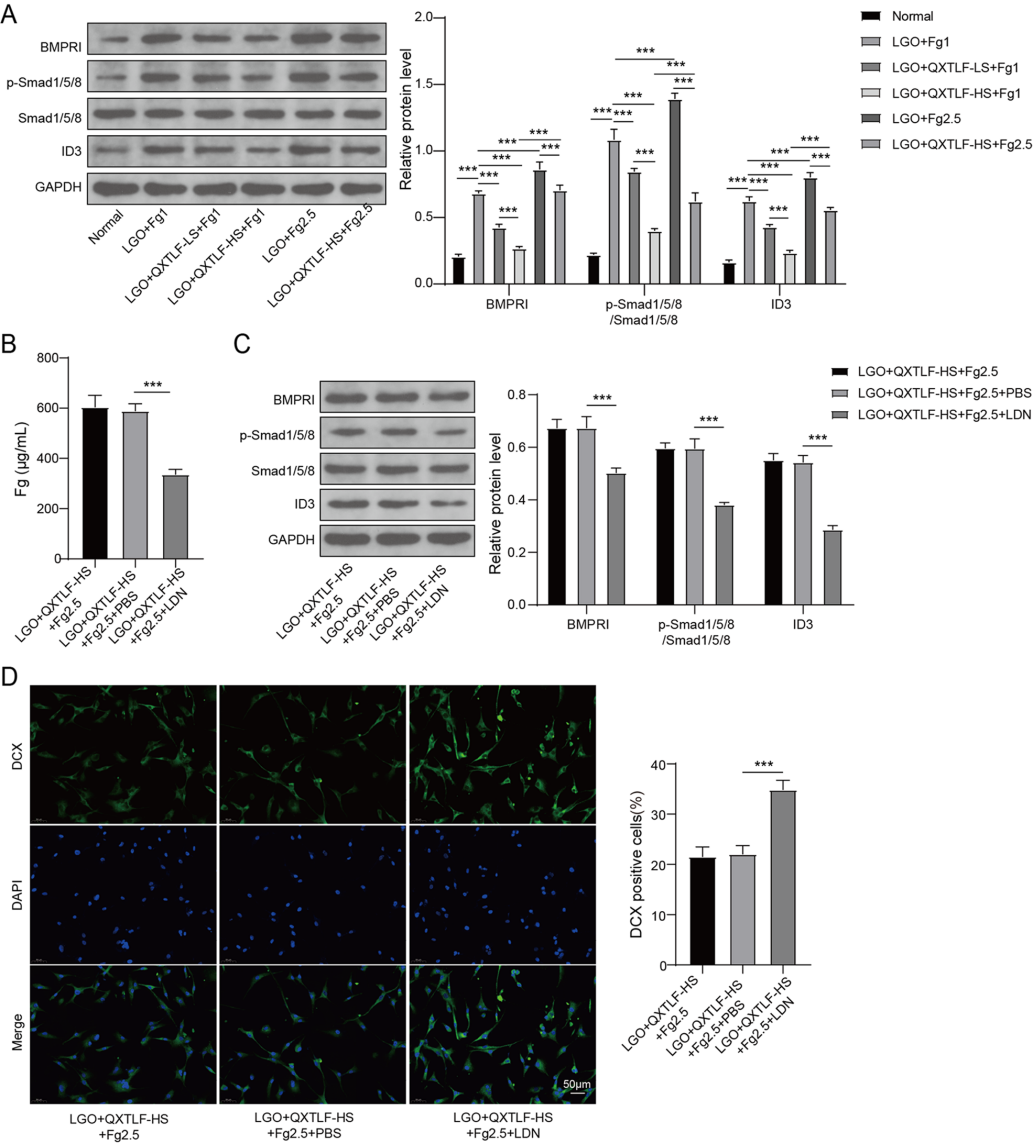

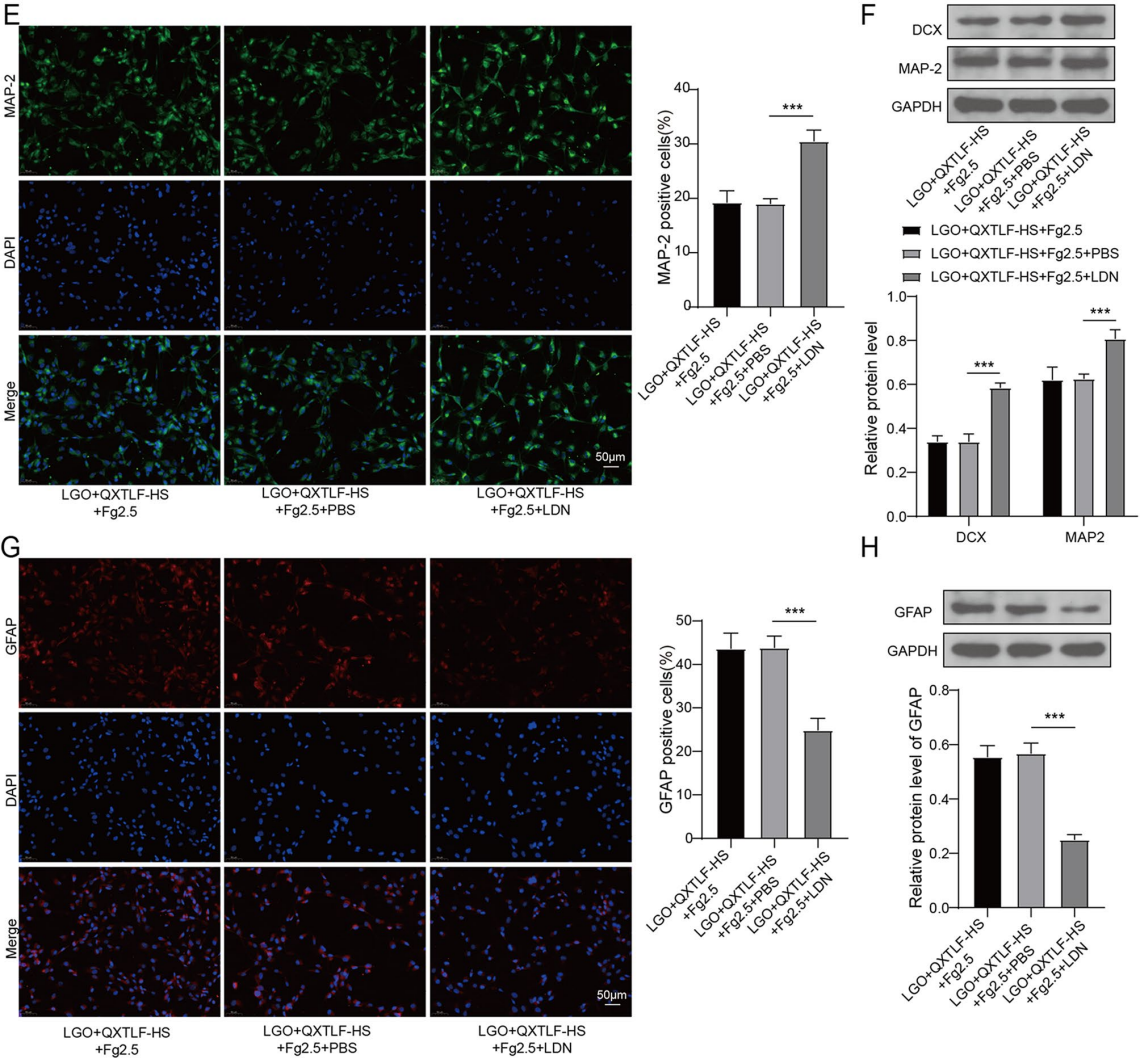
The effect of QXTLF on the regulation of the directional differentiation of LGO induced NSCs through Fg-mediated BMPRI/ID3 signal axis. (**A**) LGO induced NSCs were treated with drug-containing serum of QXTLF and/or Fg at different concentrations. Protein expressions of BMPRI, p-Smad1/5/8 and ID3 in NSCs were detected using western blot. (**B**) LGO induced NSCs were treated with QXTLF-HS, Fg2.5, and PBS or LDN. Fg content was analyzed using ELISA. (**C**) Protein expressions of BMPRI, p-Smad1/5/8 and ID3 in NSCs were quantified by western blot. (**D**) Representative image and percentage of DCX positive cells. Scale bar: 50 μm. (**E**) Representative image and percentage of MAP-2 positive cells. Scale bar: 50 μm. (**F**) Protein levels of DCX and MAP-2 were analyzed using western blot. (**G**) Representative image and percentage of GFAP positive cells. Scale bar: 50 μm. (**H**) Protein content of GFAP was detected using western blot. Data are presented as mean ± SD. ***P < 0.001

To further elucidate the mechanism underlying QXTLF-mediated regulation of LGO-induced NSCs, we investigated whether the Fg-mediated BMPRI/ID3 signaling axis is involved in their directional differentiation. LGO-induced NSCs were treated with serum containing QXTLF and/or Fg at different concentrations. LGO exposure significantly up-regulated the protein levels of BMPRI, p-Smad1/5/8 and ID3 in NSCs compared to the normal. However, treatment with serum containing QXTLF led to a dose-dependent decrease in these protein levels. Interestingly, high-dose Fg reversed the inhibitory effect of serum containing QXTLF on the levels of BMPRI, p-Smad1/5/8 and ID3 in LGO-induced NSCs (Fig. 6A). ELISA analysis demonstrated that treatment with the BMPRI kinase inhibitor LDN193189 (500 nM, Selleckchem, USA) decreased Fg content in LGO-induced NSCs treated with high-dose serum containing QXTLF and Fg (Fig. 6B). Similarly, the levels of BMPRI, p-Smad1/5/8 and ID3 were notably reduced in LGO-induced NSCs treated with high-dose QXTLF and Fg (Fig. 6C). Immunofluorescence staining further verified that LDN treatment increased the number of DCX-positive cells and MAP-2-positive cells (Fig. 6D-E), indicating that suppression of BMPRI accelerated the directional differentiation of NSCs into neurons. These findings were further confirmed by western blot analysis of DCX and MAP-2 protein levels in LGO-induced NSCs (Fig. 6F). Additionally, inhibition of BMPRI reduced the number of GFAP-positive cells in LGO-induced NSCs treated with serum containing QXTLF and Fg (Fig. 6G). Consistent with these findings, western blot analysis showed that the inhibition of BMPRI similarly affected GFAP protein expression in LGO-treated NSCs exposed to serum containing QXTLF and Fg (Fig. 6H). In conclusion, QXTLF regulated the directional differentiation of LGO-induced NSCs through Fg-mediated BMPRI/ID3 signaling axis.

## Discussion

NSCs play critical roles in the recovery from ischemic stroke and cerebrovascular disorders [32, 33]. Recent studies indicate that the impaired differentiation of NSCs into neurons adversely impacts post-stroke recovery [34, 35]. Emerging evidence suggests that TCM formulations may enhance NSCs differentiation into neurons, potentially offering therapeutic benefits for ischemic stroke. For example, certain TCM compounds, such as astragaloside IV and icariin, have been found to promote neurogenesis by activating NSCs to differentiate into neurons after ischemic stroke [36]. Similarly a study on the classic TCM compound Naoluoxintong revealed its ability to stimulate NSC differentiation into neurons, thereby improving outcomes in ischemic stroke [37]. In line with these findings, our study demonstrated that QXTLF promoted the directional differentiation of LGO induced NSCs into neurons via Fg-mediated BMPRI/ID3 signal axis in ischemic stroke.

Previous studies have shown that QXTLF exhibits restorative effects on cerebral ischemia. One earlier study indicated that QXTLF promoted the regeneration of endogenous NSCs in MCAO rats, particularly during the convalescence and sequelae stages [21]. Another study revealed that QXTLF enhanced synaptic plasticity and functional recovery in rats with cerebral infarction by modulating the brain-derived neurotrophic factor (BDNF)/tropomyosin receptor kinase B (TrkB)/cAMP response element-binding protein (CREB) signaling pathway [38]. These effects may be attributed to active components such as icariin and quercetin. Specifically, icariin drove neuronal differentiation of NSCs and increased cholinergic neurons in Alzheimer’s disease [39], while quercetin enhanced the differentiation and proliferation of NSCs by upregulating nuclear factor erythroid 2-related factor 2 (Nrf2) expression [40]. Consistent with these findings, the current study further demonstrated that QXTLF regulated directional differentiation in LGO-treated NSCs by reducing Fg deposition. Notably, the active constituent puerarin in QXTLF may mitigate hypoxia-induced NSC damage by increasing miRNA-214 [41], potentially facilitating NSC differentiation.

Recent evidence indicates that Fg deposition occurs in the brain during various neurological diseases and traumatic injuries, especially when the blood-brain barrier is disrupted, contributing to cognitive impairment and hindering brain repair [12]. Clinical studies also reveal a strong association between Fg and cardiovascular and cerebrovascular diseases. Baker et al. reported significant differences in Fg levels between men with and without ischemic heart disease [42], while increased Fg deposition has been observed in patients with atherothrombotic stroke or lacunar stroke [43]. The role of Fg in NSC differentiation in ischemic stroke is increasingly recognized. Fg deposition was observed in traumatic brain injury patients, correlating with microglial/macrophage activation and decreased neuronal density [44]. Hou et al. discovered that Fg deposition suppressed remyelination and the differentiation of oligodendrocyte progenitor cell in MCAO mouse [45]. Furthermore, Fg induced neural differentiation of stem/precursor cells through BMP receptor signaling [16]. These studies suggest that Fg deposition exacerbated ischemic stroke by dysregulating NSC differentiation. Based on these studies, we investigated the effect of QXTLF on Fg deposition. This study for the first time demonstrated that QXTLF attenuated cerebral stroke by inhibiting Fg deposition in LGO-induced NSCs.

Emerging evidence suggests that BMPRI/ID3 signaling axis is implicated in cell differentiation. Recent studies show that BMP4 induces ID1 expression, while ID3 suppresses the profibrotic activity of TGF-β2 in human trabecular meshwork cells [46]. ID2/3 is involved in mediating neurogenesis in a complementary manner, and ID proteins have the ability to induce NSCs differentiation [18]. Additionally, Fg promoted astrocytic differentiation of human glial precursor derived from induced pluripotent stem cell via BMPRI/ID3 activation [16]. Our findings have for the first time demonstrated that QXTLF modulated the directional differentiation of LGO-induced NSCs via Fg/BMPRI/ID3 signaling axis.

In conclusion, our study demonstrated that QXTLF promoted the directional differentiation of LGO-induced NSCs into neurons through modulation of the Fg-mediated BMPRI/ID3 signaling axis in cerebral stroke. These results may offer a new therapeutic target and potential treatment strategy for cerebral infarction.

## Data Availability

The datasets used or analyzed during this study can be made available from the corresponding author upon reasonable request.
